# Interorder horizontal gene transfer of *tet*(X3) between *Acinetobacter* spp. and Enterobacteriaceae

**DOI:** 10.1128/aac.01945-24

**Published:** 2025-06-23

**Authors:** Chen Xu, Xiaofan Li, Yanyan Zhang, Yuanyuan Li, Yunbing Li, Rong Zhang, Ning Dong

**Affiliations:** 1 Department of Emergency Medicine, Second Affiliated Hospital, School of Public Health, Zhejiang University School of Medicine 26441 , Hangzhou, China; 2 Department of Laboratory Medicine, School of Medicine, Jiangsu University 12676, Zhenjiang, China; 3 Department of Clinical Laboratory, Second Affiliated Hospital of Zhejiang University, Hangzhou, China; 4 Department of Medical Microbiology, Experimental Center, Suzhou Medical College, Soochow University 12582, Suzhou, China; 5 Liangzhu Laboratory, Zhejiang University 12377, Hangzhou, China; University of Fribourg, Fribourg, Switzerland

**Keywords:** tigecycline resistance, *tet*(X3), conjugation, *Acinetobacter*, Enterobactericeae

## LETTER

The clinical potential of tigecycline has been significantly challenged by the emergence of tigecycline-resistance genes, notably the plasmid-mediated *tet*(X) variants such as *tet*(X3) and *tet*(X4) encoding tigecycline-inactivating enzymes (1). *tet*(X) orthologs are distributed widely in several orders, including Flavobacteriales, Pseudomonadales, Enterobacterales, and Sphingobacteriales (2, 3). Despite this broad host range, interorder horizontal transfer of *tet*(X) was not observed previously. Herein, we reported, to our knowledge, the first *tet*(X3)-carrying plasmid transmissible between Pseudomonadales (*Acinetobacter* spp.) and Enterobacterales (Enterobacteriaceae). Furthermore, we analyzed the fitness burden, global genomic distribution, and potential species origin of this plasmid.

**Fig 1:**
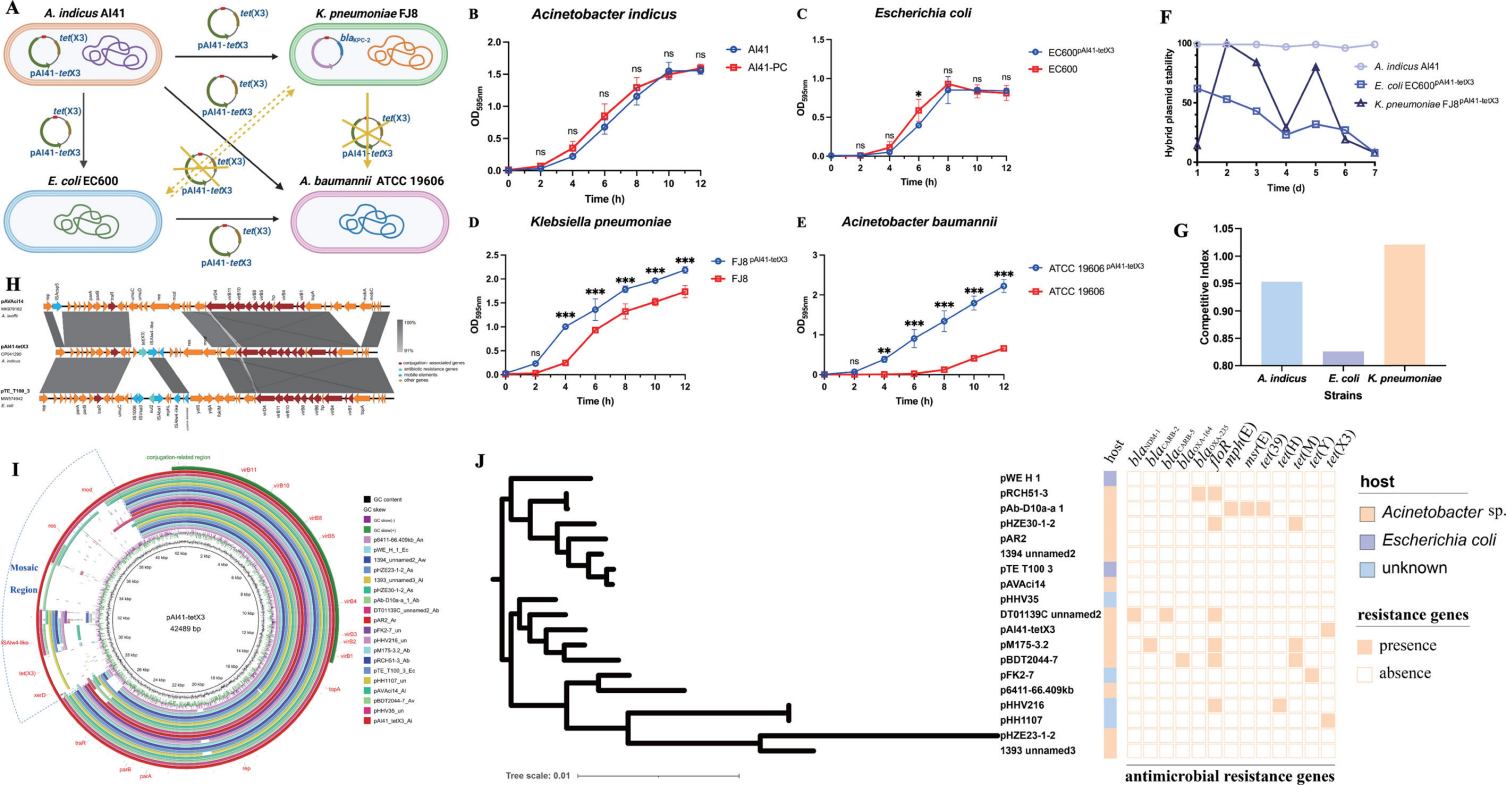
Map, transferability, fitness burden, and molecular epidemiology of pAI41-tetX3-like plasmids. (**A**) Schematic diagram depicting the conjugal transferability of pAI41-tetX3 among *Acinetobacter* sp., *Klebsiella pneumoniae,* and *Escherichia coli* strains. Blue and yellow arrows indicate that plasmid pAI41-tetX3 was conjugatively transferable and non-transferable between the two strains, respectively. (**B–E**) Growth curve of *A. indicus* (**B**), *E. coli* (**C**), *K. pneumoniae* (**D**), and *A. baumannii* (**E**) strains with and without pAI41-tetX3. The *X*-axis represents the inoculation time, and the *Y*-axis represents the optical density (OD_595_) of the culture. (**F**) Plasmid stability of pAI41-tetX3 in *A. indicus*, *E. coli,* and *K. pneumoniae* strains over 7 days. The *X*-axis represents the day of passage, and the Y-axis represents the number of colonies that grew on the LB plates supplemented with antibiotics. (**G**) *In vitro* competition results of *A. indicus*, *E. coli,* and *K. pneumoniae* strains with and without pAI41-tetX3. The *X*-axis represents the species of strain, and the *Y*-axis represents the value of the competitive index. (**H**) Linear alignment of pAI41-tetX3 with similar plasmids in the NCBI database. The original plasmid map of pAI41-tetX3 was published in a previous study (4), and panels A and B were replotted using the plasmid sequence of pAI41-tetX3 as the reference. (**I**) Circular alignment of pAI41-tetX3 with similar plasmids in the NCBI database. The original plasmid map of pAI41-tetX3 was published in a previous study (4), and panels A and B were replotted using the plasmid sequence of pAI41-tetX3 as the reference. (**J**) Mid-point rooted phylogenetic tree of pAI41-tetX3-like plasmids. The host of the plasmid (*Acinetobacter* sp., *E. coli,* or unknown) and the presence of several important antimicrobial resistance genes on the plasmids were plotted alongside the phylogenetic tree.

*Acinetobacter indicus* AI41, carrying an untypable *tet*(X3)-positive plasmid pAI41-tetX3 (length, 42,489 bp and GC content, 35.8%), was isolated from a dairy cow (4). BLAST analysis in the NCBI database on 15 September 2024 identified a total of 18 plasmid sequences that showed ≥94% identity with pAI41-tetX3 at ≥69% coverage. These plasmids were from different species, including *Acinetobacter* spp. (*n* = 12), *Escherichia coli* (*n* = 2), and uncultured bacteria (*n* = 4) isolated from four continents (Asia, Europe, Oceania, North America, and South America), suggesting their potential interorder transferability (Table S1). Results of *in vitro* conjugation suggested that pAI41-tetX3 could be transferred from AI41 to *Acinetobacter baumannii*, *E. coli,* and *Klebsiella pneumoniae* at frequencies of 1 × 10^−3^, 1 × 10^−3^, and 1 × 10^−5^, respectively (Table S2). pAI41-tetX3 in *E. coli* was also transferable to *A. baumannii* at the frequency of 5 × 10^−6^, yet pAI41-tetX3 in *E. coli* was non-conjugatively transferable to *K. pneumoniae* and vice versa (Fig. 1A). These results demonstrated the interorder transferability of pAI41-tetX3 between *Acinetobacter* spp. and Enterobacteriaceae, but its transmission potential within Enterobacteriaceae might be limited. pAI41-tetX3 was stably maintained in its original host, *A. indicus* AI41, and did not confer a significant fitness cost to it. In contrast, pAI41-tetX3 was not maintained in the Enterobacteriaceae hosts and constituted a fitness burden to *E. coli* and *K. pneumoniae* (Fig. 1B through G).

All pAI41-tetX3-like plasmids shared a backbone of approximately 27 kb, which harbored the conjugation-associated cassette. Apart from six plasmids in *Acinetobacter* spp. that did not carry resistance genes, a dynamic mosaic region carrying 1–7 resistance genes and diverse mobile genetic elements was observed on other plasmids, suggesting this region was a hot spot for genetic recombination, which might drive the constant evolution of pAI41-tetX3-like plasmids (Fig. 1G through I; Fig. S1). The average GC content of pAI41-tetX3-like plasmids was 38.1% (range 34.95%–43.11%), similar to that of the *Acinetobacter* population (average 39.6%, range 37%–46%) (Table S1). The proximity in GC content, the presence of the prototype plasmid (pAI41-tetX3-like plasmids carrying no resistance gene), the stability, and low fitness burden of pAI41-tetX3 in *Acinetobacter*, all suggest that such plasmids could have originated from an *Acinetobacter* species and transferred to Enterobacteriaceae. In addition, the results of codon bias analysis showed that pAI41-tetX3 was more similar to *A. baumannii* ATCC 19606, followed by *A. indicus* AI41 and *E. coli* EC600 (Table S5).

In conclusion, we report a *tet*(X3)-carrying plasmid conferring tigecycline resistance, potentially originating from an *Acinetobacter* species, and capable of interorder horizontal transfer between *Acinetobacter* spp. and Enterobacteriaceae. The global dissemination, potential for constant evolution, and transferability of such resistance plasmids pose a significant public threat.

## SUPPLEMENTAL MATERIAL

Supplemental material aac.01945-24-s0001.docx Fig. S1; Tables S1 to S5.

ASM does not own the copyrights to Supplemental Material that may be linked to, or accessed through, an article. The authors have granted ASM a non-exclusive, world-wide license to publish the Supplemental Material files. Please contact the corresponding author directly for reuse.
