# Supplementary material for: Interorder horizontal gene transfer of tet(X3) between Acinetobacter spp. and Enterobacteriaceae
Source: Antimicrob Agents Chemother. 2025 Jun 23;69(8):e01945-24. doi: 10.1128/aac.01945-24 (PMC12326979; doi:10.1128/aac.01945-24)
Supplement: Supplemental material — Fig. S1; Tables S1 to S5. [file aac.01945-24-s0001.docx]

**Supplementary Materials**

**1. Supplementary Methods**

**1.1. Strain source**

*A. indicus* strain AI41 carrying plasmid-borne *tet*(X3) genes was recovered from the rectal swab sample from a dairy cow in a Chinese farm as previously described (Zhang et al., 2020b). This strain was subjected to further genomic and phenotypic characterization in this study. Recipients used in the conjugation assay including rifampicin-resistant *A. baumannii* ATCC 19606, rifampicin-resistant *E. coli* EC600, carbapenem-resistant *K. pneumoniae* FJ8 and rifampicin-resistant *K. pneumoniae* ATCC 4352 were strains preserved in our laboratory.

**1.2. Plasmid sequence acquisition and bioinformatics analyses**

The complete sequence of plasmid pAI41-tetX3 (GenBank accession: CP041290) was obtained previously (Zhang et al., 2020b). Plasmids that shared similar backbone with pAI41-tetX3 was obtained by BLASTn search in the NCBI nr/nt database, and only plasmid sequences that showed >93% identities at >65% coverages were used in this study. Antimicrobial resistance genes in all plasmids were analyzed with ResFinder 4.1 (Kleinheinz et al., 2014). Circular and linear comparison of plasmid sequences was conducted using BRIG v0.95 and Easyfig v2.1, respectively (Alikhan et al., 2011; Sullivan et al., 2011). All plasmid sequences were annotated with Prokka v1.13 and the generated gff files were further analyzed with the pan genome pipeline Roary v3.13.0 (Page et al., 2015; Seemann, 2014). The phylogenetic tree was built with FastTree 2 based on the multiple core gene alignment (concatenated core genes) file, which was the output file from Roary (Price et al., 2010). The tree was visualized and edited with iTOL v6 (Letunic and Bork, 2021).

**1.3. Antimicrobial susceptibility testing**

The antimicrobial resistance susceptibility of all strains in this study to 16 commonly used antibiotics including ampicillin, ampicillin-sulbactam, piperacillin-tazobactam, ceftazidime, cefepime, aztreonam, imipenem, meropenem, amikacin, gentamicin, tobramycin, ciprofloxacin, levofloxacin, sulfamethoxazole-trimethoprim, oxytetracycline and tigecycline was tested using the broth dilution method. *E. coli* ATCC 25922 was tested as the quality control. The results were interpreted according to the CLSI guideline, except that for tigecycline, which was interpreted in accordance with the EUCAST breakpoints (Testing and Testing, 2021; Weinstein, 2021).

**1.4. Conjugation assay**

The interspecies transferability of pAI41-tetX3 between *Acinetobacter* species and Enterobacteriaceae was evaluated by conjugation experiment using the filter-mating method. Firstly, *A. indicus* AI41 was used as the donor and *A. baumannii* ATCC 19606 (Rif^R^), *E. coli* EC600 (Rif^R^), *K. pneumoniae* FJ8 (Mem^R^) and *K. pneumoniae* ATCC 4352 (Rif^R^) were used as the recipients in defferent temperatures. Transconjugants were selected on LB agar plates containing 0.5 mg/L tigecycline with 600 mg/L rifampicin, 600 mg/L rifampicin, 1 mg/L meropenem or 600 mg/L rifampicin. Successful transconjugants were termed *A. baumannii* ATCC 19606^pAI41-tetX3^, *E. coli* EC600^pAI41-tetX3^, *K. pneumoniae* FJ8^pAI41-tetX3^ and *K. pneumoniae* ATCC 4352^pAI41-tetX3^ respectively. The transconjugants from the first conjugation assay were further used as donors of the second conjugation assay to further test the transferability of pAI41-tetX3. Briefly, we tested the transferability of pAI41-tetX3 from *K. pneumonia* FJ8^pAI41-tetX3^ to *A. baumannii* ATCC 19606 (Rif^R^) and *E. coli* EC600 (Rif^R^), and the transconjugants were selected on LB agar plates containing 0.5 mg/L tigecycline with 600 mg/L rifampicin. Also, the transferability of pAI41-tetX3 from EC600^pAI41-tetX3^ to *A. baumannii* ATCC 19606 (Rif^R^) and *K. pneumoniae* FJ8 (Mem^R^) were tested with the transconjugants were selected on LB agar plates containing 0.5 mg/L tigecycline with either 600 mg/L rifampicin, or 1 mg/L meropenem, respectively. Conjugative frequency were calculated as the ratio between the number of transconjugants obtained from each specific condition and the number of donors.

The species of the transconjugants were verified by MALDI-TOF MS. To prove the complete plasmid pAI41-tetX3 transferred to the recipients rather than only the mobile fragment carrying *tet*(X3), plasmids in successful transconjugants were extracted and sequenced with Oxford Nanopore MinION. The raw sequences were assembled with Canu 1.6 (Koren et al., 2017). The presence of pAI41-tetX3 was validated by aligning the obtained sequence with the sequence of pAI41-tetX3.

**1.5. Plasmid elimination assay**

Elimination of pAI41-tetX3 from *A. indicus* AI41 was performed using SDS as previously described (El-Mansi et al., 2000). Briefly, single colonies of strain AI41 were picked from LB agar plates and inoculated into LB broth at 37°C overnight. Subsequently, a volume of 100 μL of bacterial culture was added to fresh LB broth containing 5%, 4%, 3%, 2% and 1% SDS, and incubated at 27°C overnight. Then serially diluted overnight cultures were spread on LB plates. Single colonies were streaked simultaneously on drug-free LB plates and LB plates supplemented with 0.25 mg/L tigecycline. Colonies that grew on drug-free LB plates but not on the plates with antibiotics were collected and plasmid elimination was further conﬁrmed by PCR using primers described previously (He et al., 2019).

**1.6. Growth rate assay**

The growth rate assay of all tested strains was conducted using methods described previously (Liu et al., 2022). Briefly, overnight cultures of all strains were subcultured 1:1000. Grow the strains at 37 ℃ with agitation at 200 rpm in triplicate. Bacterial growth was monitored at 2 h time intervals for 12 h by measuring the optical density at 595 nm (OD_595_) using a SpectraMax Plus microplate reader (Molecular Devices, San Jose, CA, USA). Growth curves were plotted using GraphPad Prism v 8.0.1 (GraphPad, San Diego, CA). The growth rate was calculated using methods described previously (Pérez et al., 2012).

**1.7. Plasmid stability tests**

The stability of plasmid pAI41-tetX3 in different bacterial species was evaluated by passaging on LB agar plates for 7 days (Sun et al., 2016). Briefly, 100 independent lineages for each of *A. indicus* AI41, *E. coli* EC600^pAI41-tetX3^ and *K. pneumoniae* FJ8^pAI41-tetX3^ were tested by single colony streaking on LB plates without antibiotic. Loss of plasmid was assessed by cross-streaking on LB plates without antibiotic and LB plates supplemented with tigecycline (0.5mg/L) and verified by PCR targeting *tet*(X3).

**1.8. *In vitro* competition assay**

Competition assay was performed as previously described to evaluate the *in vitro* relative growth advantage between *A. indicus*, *E. coli* and *K. pneumoniae* strains with or without plasmid pAI41-tetX3 (Liu et al., 2022; Nang et al., 2018). Briefly, each pair of strains, i.e., *A. indicus* AI41 and AI41-PC, *E. coli* EC600 and EC600^pAI41-tetX3^, and *K. pneumoniae* FJ8 and FJ8^pAI41-tetX3^ were mixed in a 1:1 ratio at 0 h in 5 mL LB broth. The cultures were grown at 37°C for 24 h with shaking at 200 rpm in duplicate. Dilutions of the mixture at 0 and 24 h were spread on both drug-free LB agar plates and LB agar plates supplemented with 0.25 mg/L tigecycline to determine the number of colonies of tested strains. The competitive index ( CI ) value was calculated by the ratio of the average CFU count of the plasmid-carrying strain to the average CFU count of the parent strain without the plasmid. A competitive index value of <1 indicates a fitness defect and a value of >1 indicates a ﬁtness beneﬁt.

**1.9. Comparison of codon preference**

The frequency of codon occurrence is expressed in thousandths as the frequency of usage of a certain codon in the total number of codons encoding each protein coding sequence. Codon W software was used to calculate the frequency of codon usage in plasmids and strains. The ratio of the frequency of codon usage in the plasmid to the frequency of each codon in other strains was calculated. The ratio between 0.5 and 2.0 indicated that the two species had similar preferences for the codon, and vice versa(Lu et al., 2023).

**2. Supplementary Discussion**

Horizontal gene transfer (HGT), involving mechanism such as conjugation, transformation, transduction, and vesiduction, plays a pivotal role in the dissemination of antimicrobial resistance (Soler and Forterre, 2020). Among these, plasmid-mediated conjugative transfer is the most superior mechanism of HGT and has extensively contributed to the acquisition and transfer of antimicrobial resistance genes (Pu et al., 2021). Conjugation is a process in which plasmid DNA is transferred from the donor to the recipient bacterium by direct contact, enabling bacteria to modify their metabolic properties by exchanging genetic contents (Wang et al., 2021).

The host range of a plasmid is crucial to its persistence and dissemination in bacterial communities (Yang et al., 2024). The *tet*(X) orthologues, which were proposed to originate from Flavobacteriaceae (a genus from the Bacteroidota phylum), distributed in different families including Moraxellaceae, Flavobacteriaceae, Enterobacteriaceae, Pseudomonadaceae and Sphingobacteriaceae (Dong et al., 2022; Zhang et al., 2020a). This situation suggested that the inter-family transfer of *tet*(X) genes could occur spontaneously, albeit at a relatively rare frequency. According to previous studies, plasmids found in *Acinetobacter* spp. were largely confined to the *Acinetobacter* genus, i.e., the plasmids found in other Gram-negative species, especially Enterobacterales, do not appear to be stably maintained in *Acinetobacter* species and *Acinetobacter* plasmids are not seen in other Gram-negative pathogens (Lam et al., 2023). To our knowledge, this is the first study reporting the transferability of a *tet*(X)-carrying plasmid between *Acinetobacter* spp. and Enterobacteriaceae, which shed light on the understanding of the widespread dissemination of this last-line antibiotic resistance gene. Besides, pAI41-tetX-like plasmids could be promising tools for the investigation of the co-evolution of the *Acinetobacter* spp. and Enterobacteriaceae driven by plasmids.

Plasmids are often metabolically burdensome to their bacterial hosts, resulting in an observable fitness cost which dictates the extent and trajectory of plasmid spread in microbial environments (Ahmad et al., 2023). We found that the transferable plasmid pAI41-tetX3 was stably maintained in its natural host, *A. indicus* strain AI41, and did not confer a signiﬁcant ﬁtness cost to strain AI41. In contrast, pAI41-tetX3 was not stably maintained in the Enterobacteriaceae hosts and constituted a ﬁtness burden to *E. coli* and *K. pneumoniae*. Genetic analysis suggested the *tet*(X3)-carrying plasmid, pAI41-tetX3, in this study could have originated from an *Acinetobacter* strain and transferred to Enterobacteriaceae. The fitness cost could be one of the reasons that plasmids in *Acinetobacter* spp. do not resemble those found in other Gram-negative pathogens. Besides, pAI41-tetX3-like plasmids harbored a mosaic region which was a host spot for genetic recombination, generating multidrug resistant plasmids carrying diverse resistance genes. Future research should focus on the development of intervention measures to prevent further dissemination of such plasmids.


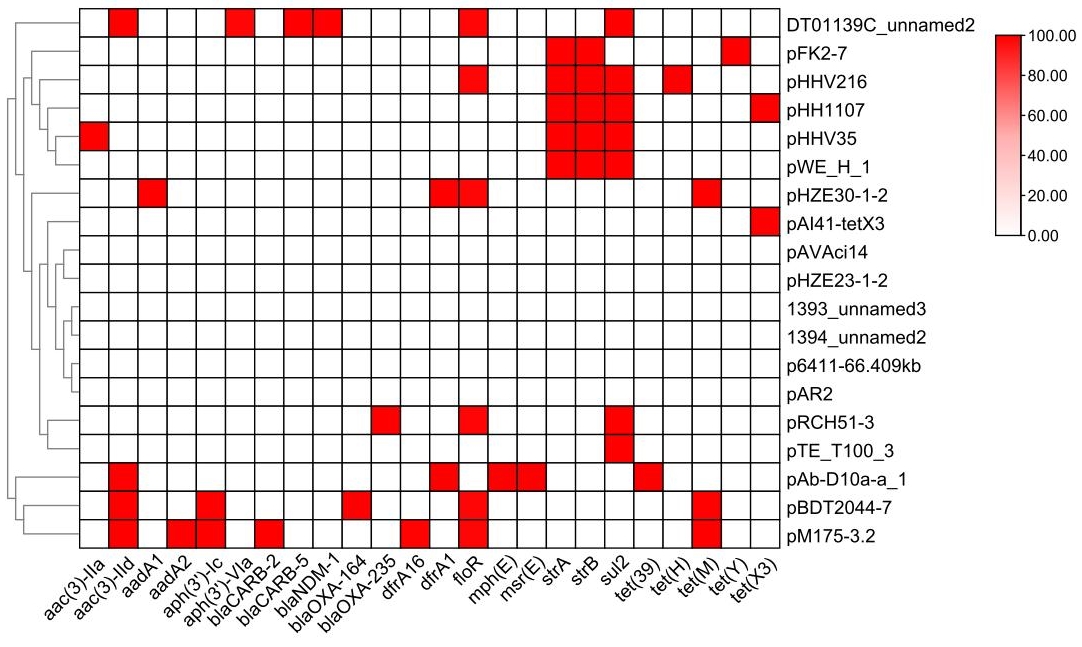


**Supplementary Figure 1. Heatmap of antimicrobial resistance genes carried by pAI41-tetX3-like plasmids.** Horizontal and vertical axes represent the antimicrobial resistance genes and the plasmid IDs, respectively. Red and white boxes represent the presence and absence of the corresponding items among sequenced isolates, respectively. The gradient identity bar indicates the percentage similarity of the related gene. The similarity dendrogram was calculated using agglomerative hierarchical clustering, with the degree of similarity between different clusters being calculated by the average linkage method and the degree of similarity of different isolates calculated with Spearman’s rank correlation coefﬁcient.

**Supplementary Table 1. Information of plasmids sharing similar backbone with pAI41-tetX3 in the NCBI database.**

| Plasmid ID | Species | Max Score | Total Score | Query Cover | E value | Per. ident | Acc. Len | Accession | GC content | Country of origin |
| --- | --- | --- | --- | --- | --- | --- | --- | --- | --- | --- |
| pHHV35 | uncultured bacterium HHV35 | 47256 | 76530 | 89% | 0 | 99.03 | 59113 | FJ012882.1 | 41.30% | Germany |
| pBDT2044-7 | *Acinetobacter variabilis* | 26548 | 71519 | 85% | 0 | 99.45 | 55901 | CP094253.1 | 38.39% | China |
| pAVAci14 | *Acinetobacter lwoffii* | 28605 | 68826 | 84% | 0 | 99.36 | 45741 | MK978162.1 | 34.95% | Hungary |
| pHH1107 | uncultured bacterium HH1107 | 24354 | 62577 | 81% | 0 | 97.61 | 57951 | FJ012881.1 | 41.47% | Germany |
| pTE_T100_3 | *Escherichia coli* | 34278 | 67161 | 78% | 0 | 99.39 | 46853 | MW574942.1 | 36.38% | Sweden |
| pRCH51-3 | *Acinetobacter baumannii* | 22306 | 61701 | 77% | 0 | 99.2 | 52789 | KY216144.1 | 37.71% | Australia |
| pM175-3.2 | *Acinetobacter baumannii* | 46817 | 64097 | 76% | 0 | 99.28 | 57742 | CP059476.1 | 38.32% | China |
| pHHV216 | uncultured bacterium HHV216 | 24354 | 58952 | 76% | 0 | 97.61 | 58274 | FJ012880.1 | 41.39% | Germany |
| pFK2-7 | uncultured bacterium | 34583 | 60689 | 74% | 0 | 98.8 | 48493 | KT325596.1 | 37.45% | Czech Republic |
| pAR2 | *Acinetobacter radioresistens* | 49783 | 58352 | 73% | 0 | 98.8 | 69207 | CP038024.1 | 36.98% | Chile |
| DT01139C unnamed2 | *Acinetobacter baumannii* | 34343 | 61825 | 73% | 0 | 99.43 | 63650 | CP053220.1 | 43.11% | Norway |
| pAb-D10a-a_1 | *Acinetobacter baumannii* | 34228 | 60031 | 73% | 0 | 99.34 | 48239 | CP051870.1 | 37.08% | Japan |
| pHZE30-1-2 | *Acinetobacter schindleri* | 19874 | 67306 | 73% | 0 | 99.18 | 68525 | CP044485.1 | 39.29% | China |
| 1393 unnamed3 | *Acinetobacter lwoffii* | 17547 | 54073 | 73% | 0 | 98.2 | 55306 | CP077339.1 | 39.06% | USA |
| pHZE23-1-2 | *Acinetobacter schindleri* | 13553 | 54938 | 73% | 0 | 94.68 | 34862 | CP044465.1 | 36.61% | China |
| 1394 unnamed2 | *Acinetobacter lwoffii* | 28354 | 57249 | 72% | 0 | 99.13 | 47918 | CP077371.1 | 35.14% | USA |
| pWE_H_1 | *Escherichia coli* | 41092 | 57564 | 70% | 0 | 98.89 | 37747 | MW574947.1 | 37.61% | Sweden |
| p6411-66.409kb | *Acinetobacter nosocomialis* | 34747 | 56233 | 69% | 0 | 98.34 | 66409 | CP010903.1 | 36.36% | USA |

Note: 0 in the column of E values is the output when the precision is insufficient (< 0.5×10^-324^).

**Supplementary Table 2. Antimicrobial susceptibility profiles of strains in this study**

| Strain | Species | Type | Conjugation | Minimum Inhibitory Concentration (mg/L) | | | | | | | | | | | | | | | | |
| --- | --- | --- | --- | --- | --- | --- | --- | --- | --- | --- | --- | --- | --- | --- | --- | --- | --- | --- | --- | --- |
|  |  |  | frequency | AMP | SAM | TZP | CAZ | FEP | ATM | IMP | MEM | AMK | GM | TOB | CIP | LEV | SXT | OXY | TGC | RIF |
| AI41 | *Acinetobacter indicus* | donor | - | ≤2 | ≤2 | ≤4 | ≤1 | ≤1 | 2 | ≤1 | ≤0.06 | ≤2 | ≤1 | ≤1 | ≤0.25 | ≤0.25 | ≤20 | 64 | 1 | <32 |
| AI41-PC | *Acinetobacter indicus* | plasmid eliminated | - | ≤2 | ≤2 | ≤4 | ≤1 | ≤1 | ≤1 | ≤1 | ≤0.06 | ≤1 | ≤0.25 | ≤0.25 | ≤0.25 | ≤0.25 | ≤20 | 0.5 | ≤0.06 | <32 |
| EC600 | *Escherichia coli* | recipient | - | 16 | 8 | ≤4 | ≤1 | ≤1 | ≤1 | ≤1 | ≤0.06 | ≤2 | ≤1 | ≤1 | ≤0.25 | 0.5 | ≤20 | 4 | 0.125 | >2400 |
| EC600^pAI41-tetX3^ | *Escherichia coli* | transconjugant | 1×10^-3^ | 16 | 8 | ≤4 | ≤1 | ≤1 | ≤1 | ≤1 | ≤0.06 | ≤2 | ≤1 | ≤1 | ≤0.25 | 0.5 | ≤20 | 32 | 1 | >2400 |
| ATCC 19606 | *Acinetobacter baumannii* | recipient | - | >32 | >32 | >128 | ≤1 | ≤1 | ≤1 | ≤1 | 0.25 | ≤2 | ≤1 | ≤1 | ≤0.25 | ≤0.25 | ≤20 | 8 | 0.125 | >2400 |
| ATCC 19606 ^pAI41-tetX3^ | *Acinetobacter baumannii* | transconjugant | 1×10^-3^/5×10^-6^ * | >32 | ≤2 | >128 | 8 | ≤1 | ≤1 | 2 | ≤0.06 | ≤1 | 1 | 1 | ≤0.25 | ≤0.25 | ≤20 | 128 | 8 | >2400 |
| FJ8 | *Klebsiella pneumoniae* | recipient | - | >32 | >32 | >128 | >64 | >64 | >64 | >16 | >32 | >64 | >16 | >16 | >4 | >8 | ≤20 | 16 | 2 | <32 |
| FJ8 ^pAI41-tetX3^ | *Klebsiella pneumoniae* | transconjugant | 1×10^-5^ | >32 | >32 | >128 | >64 | >64 | >64 | >16 | >32 | >64 | >16 | >16 | >4 | >8 | ≤20 | 64 | 4 | <32 |

* The two numbers represent the frequencies of conjugation from *A. indicus* AI41 to *A.* *baumannii*ATCC 19606 and from *E. coli* EC600 to *A.* *baumannii*ATCC 19606, respectively.

Abbreviations: AMP, ampicillin; SAM, ampicillin/sulbactam; TZP, piperacillin/tazobactam; CAZ, ceftazidime; FEP, cefepime; ATM, aztreonam; IMP, imipenem; MEM, meropenem; AMK, amikacin; GM, gentamicin; TOB, tobramycin; CIP, ciprofloxacin; LEV, levofloxacin; SXT, sulfamethoxazole/trimethoprim; OXY, oxytetracycline; TGC, tigecycline; RIF, Rifampicin.

**Supplementary Table 3. Conjugative transfer frequency from AI41 to EC600, ATCC 19606, FJ8, ATCC 4352 upon treatment with different mating temperatures.**

| Strain | Species | Type | Conjugation temperature (℃) | Conjugation frequency |
| --- | --- | --- | --- | --- |
| EC600 ^pAI41-tetX3^ | *Escherichia coli* | transconjugant | 30 | 2×10^-1^ |
|  |  |  | 37 | 5×10^-1^ |
|  |  |  | 42 | 9×10^-3^ |
| ATCC 19606 ^pAI41-tetX3^ | *Acinetobacter baumannii* | transconjugant | 30 | 5×10^-3^ |
|  |  |  | 37 | 3×10^-4^ |
|  |  |  | 42 | 4×10^-5^ |
| FJ8 ^pAI41-tetX3^ | *Klebsiella pneumoniae* | transconjugant | 30 | 4×10^-5^ |
|  |  |  | 37 | 5×10^-5^ |
|  |  |  | 42 | 1×10^-6^ |
| ATCC 4352 ^pAI41-tetX3^ | *Klebsiella pneumoniae* | transconjugant | 30 | 1×10^-4^ |
|  |  |  | 37 | 1×10^-3^ |
|  |  |  | 42 | 9×10^-5^ |

**Supplementary Table 4. Conjugative transfer frequency from AI41 to EC600, ATCC 19606, FJ8, ATCC 4352 upon treatment in different mating temperatures.**

| strain | Number of plasmids | Plasmid type | integrative conjugative elements | resistance gene |
| --- | --- | --- | --- | --- |
| *A. indicus* AI41 | 1 | non-typable | IS*AIw4* | *tet*(X3) |
| *E. coli* EC600 ^RifR^ | 1 | IncL/M(pMU407) | IS*3000*、IS*Kpn19*、IS*Kpn6*、IS*Kpn27*、IS*26*、Tn3、IS*Swi1* | *bla*_KPC-2_、*qrS1* |
| *A. baumannii* ATCC 19606 ^RifR^ | 0 | - | - | - |
| *K. pneumoniae* FJ8^MemR^ | 4 | IncFII(pHN7A8)、IncR | IS*1R*、Tn2、IS*26*、IS*Ecp1*、IS*903B*、IS*1294*、IS*1R*、IS*Kpn6*、IS*Kpn27*、IS*5075*、IS*Kpn28*、IS*Kpn26*、TnAs2、 | *bla*_CTX-M_、*fosA*、*bla*_TEM_、*bla*_KPC-2_、*catA2* |
|  |  | IncFII(pECLA)、IncI1 | IS*26* | - |
|  |  | ColRNAI | - | - |
|  |  | ColRNAI | - | - |
| *K. pneumoniae* ATCC 4352 ^RifR^ | 0 | - | - | - |

**Supplementary Table 5. Comparison of codon usage frequency**

| amino acid | codon | plasmid pAI41-tetX3 | *A. indicus* AI41 | *A. baumannii* ATCC 19606 | *E. coli* EC600 | *K. pneumoniae* FJ8 | *K. pneumoniae*  ATCC 4352 | plasmid pAI41-tetX3/ *A. indicus* AI41 | plasmid pAI41-tetX3/  *A. baumannii* ATCC 19606 | plasmid pAI41-tetX3/  *E. coli* EC600 | plasmid pAI41-tetX3/  *K. pneumoniae* FJ8 | plasmid pAI41-tetX3/  *K. pneumoniae* ATCC 4352 |
| --- | --- | --- | --- | --- | --- | --- | --- | --- | --- | --- | --- | --- |
| Phe | UUU | 50.1 | 33.4 | 40.5 | 23.5 | 17.1 | 17.0 | 1.50 | 1.24 | 2.13 | 2.93 | 2.94 |
|  | UUC | 22.8 | 19.6 | 18.8 | 18.2 | 15.4 | 14.8 | 1.16 | 1.21 | 1.25 | 1.48 | 1.54 |
| Leu | UUA | 34.7 | 18.9 | 27.4 | 14.9 | 9.9 | 10.0 | 1.83 | 1.27 | 2.34 | 3.50 | 3.49 |
|  | UUG | 21.7 | 20.7 | 25.1 | 16.3 | 11.4 | 11.4 | 1.05 | 0.87 | 1.33 | 1.91 | 1.90 |
|  | CUU | 23.7 | 17.1 | 21.1 | 13.4 | 12.1 | 12.0 | 1.39 | 1.13 | 1.77 | 1.96 | 1.97 |
|  | CUC | 6.1 | 7.7 | 8.7 | 9.1 | 11.9 | 11.6 | 0.80 | 0.71 | 0.67 | 0.52 | 0.53 |
|  | CUA | 15.3 | 7.7 | 11.3 | 5.8 | 5.2 | 5.5 | 1.98 | 1.35 | 2.61 | 2.93 | 2.79 |
|  | CUG | 15.1 | 23.5 | 14.3 | 22.4 | 25.2 | 25.7 | 0.64 | 1.05 | 0.68 | 0.60 | 0.59 |
| Ile | AUU | 31.5 | 25.4 | 30.4 | 17.9 | 11.7 | 11.8 | 1.24 | 1.04 | 1.76 | 2.69 | 2.68 |
|  | AUC | 19.3 | 19.0 | 16.3 | 18.8 | 19.0 | 18.7 | 1.02 | 1.19 | 1.03 | 1.02 | 1.03 |
|  | AUA | 26.1 | 17.4 | 21.6 | 13.7 | 11.5 | 11.0 | 1.50 | 1.21 | 1.91 | 2.28 | 2.38 |
| Met | AUG | 15.1 | 18.9 | 19.0 | 16.4 | 14.5 | 14.2 | 0.80 | 0.80 | 0.92 | 1.04 | 1.06 |
| Val | GUU | 18.8 | 16.6 | 19.3 | 17.8 | 13.0 | 13.2 | 1.13 | 0.97 | 1.05 | 1.45 | 1.42 |
|  | GUC | 7.3 | 11.1 | 9.0 | 11.9 | 13.9 | 13.6 | 0.65 | 0.81 | 0.61 | 0.52 | 0.54 |
|  | GUA | 10.0 | 11.6 | 14.3 | 11.3 | 8.9 | 8.8 | 0.86 | 0.70 | 0.88 | 1.12 | 1.13 |
|  | GUG | 7.6 | 13.8 | 11.7 | 14.1 | 12.8 | 13.3 | 0.55 | 0.64 | 0.54 | 0.59 | 0.57 |
| Ser | UCU | 18.8 | 13.4 | 15.0 | 12.4 | 11.1 | 11.0 | 1.40 | 1.25 | 1.51 | 1.69 | 1.71 |
|  | UCC | 9.7 | 10.5 | 8.2 | 12.3 | 14.1 | 13.4 | 0.92 | 1.19 | 0.79 | 0.68 | 0.72 |
|  | UCA | 26.1 | 20.7 | 20.9 | 18.0 | 16.0 | 15.6 | 1.26 | 1.25 | 1.44 | 1.63 | 1.67 |
|  | UCG | 7.1 | 10.2 | 9.4 | 15.2 | 18.4 | 18.3 | 0.70 | 0.76 | 0.47 | 0.39 | 0.39 |
| Pro | CCU | 10.4 | 11.4 | 9.7 | 11.0 | 13.7 | 13.3 | 0.91 | 1.07 | 0.94 | 0.76 | 0.78 |
|  | CCC | 7.0 | 8.4 | 6.0 | 10.4 | 14.9 | 14.1 | 0.84 | 1.16 | 0.67 | 0.47 | 0.49 |
|  | CCA | 15.3 | 20.2 | 15.8 | 17.9 | 19.3 | 19.3 | 0.75 | 0.97 | 0.85 | 0.79 | 0.79 |
|  | CCG | 5.5 | 13.8 | 6.9 | 18.6 | 26.9 | 26.6 | 0.40 | 0.80 | 0.30 | 0.21 | 0.21 |
| Thr | ACU | 13.6 | 12.3 | 14.9 | 10.8 | 8.0 | 7.6 | 1.11 | 0.91 | 1.26 | 1.70 | 1.78 |
|  | ACC | 10.0 | 16.8 | 13.9 | 16.1 | 17.5 | 16.7 | 0.59 | 0.72 | 0.62 | 0.57 | 0.60 |
|  | ACA | 14.5 | 14.5 | 15.8 | 12.5 | 9.8 | 9.2 | 1.00 | 0.92 | 1.16 | 1.48 | 1.57 |
|  | ACG | 8.7 | 10.5 | 9.9 | 15.7 | 15.1 | 14.8 | 0.83 | 0.87 | 0.55 | 0.57 | 0.58 |
| Ala | GCU | 14.7 | 17.6 | 16.2 | 17.9 | 21.8 | 22.0 | 0.83 | 0.90 | 0.82 | 0.67 | 0.67 |
|  | GCC | 7.3 | 18.4 | 10.2 | 20.0 | 29.9 | 29.2 | 0.40 | 0.71 | 0.36 | 0.24 | 0.25 |
|  | GCA | 13.7 | 20.4 | 18.0 | 20.5 | 19.9 | 20.2 | 0.67 | 0.76 | 0.67 | 0.69 | 0.68 |
|  | GCG | 5.3 | 13.4 | 8.2 | 24.7 | 33.2 | 34.8 | 0.40 | 0.65 | 0.21 | 0.16 | 0.15 |
| Tyr | UAU | 24.4 | 17.4 | 21.2 | 13.6 | 11.0 | 11.2 | 1.40 | 1.15 | 1.78 | 2.22 | 2.17 |
|  | UAC | 11.1 | 11.6 | 14.2 | 11.4 | 9.2 | 8.7 | 0.95 | 0.78 | 0.97 | 1.20 | 1.27 |
| TER | UAA | 28.2 | 19.6 | 27.2 | 14.8 | 9.9 | 10.2 | 1.44 | 1.04 | 1.90 | 2.85 | 2.78 |
|  | UAG | 16.6 | 7.9 | 11.4 | 5.8 | 5.5 | 5.4 | 2.09 | 1.46 | 2.88 | 3.01 | 3.07 |
| His | CAU | 17.5 | 19.7 | 18.7 | 16.3 | 14.8 | 14.5 | 0.89 | 0.94 | 1.08 | 1.19 | 1.21 |
|  | CAC | 10.9 | 14.9 | 11.8 | 14.4 | 13.8 | 13.1 | 0.74 | 0.92 | 0.76 | 0.79 | 0.84 |
| Gln | CAA | 22.3 | 20.9 | 24.8 | 16.4 | 11.4 | 11.7 | 1.07 | 0.90 | 1.36 | 1.96 | 1.91 |
|  | CAG | 14.1 | 24.1 | 14.0 | 22.1 | 26.0 | 25.3 | 0.58 | 1.00 | 0.64 | 0.54 | 0.56 |
| Asn | AAU | 32.2 | 26.7 | 30.3 | 17.7 | 12.0 | 11.6 | 1.21 | 1.06 | 1.82 | 2.69 | 2.77 |
|  | AAC | 18.0 | 16.7 | 19.6 | 17.8 | 13.5 | 12.9 | 1.08 | 0.92 | 1.01 | 1.33 | 1.39 |
| Lys | AAA | 43.5 | 32.9 | 41.1 | 23.8 | 17.3 | 16.7 | 1.32 | 1.06 | 1.83 | 2.51 | 2.60 |
|  | AAG | 21.5 | 16.3 | 21.1 | 13.4 | 11.8 | 11.8 | 1.32 | 1.02 | 1.60 | 1.82 | 1.82 |
| Asp | GAU | 19.1 | 18.7 | 16.2 | 18.8 | 18.5 | 18.7 | 1.02 | 1.18 | 1.02 | 1.04 | 1.02 |
|  | GAC | 8.2 | 11.3 | 8.9 | 11.8 | 13.5 | 13.8 | 0.73 | 0.92 | 0.69 | 0.61 | 0.59 |
| Glu | GAA | 18.6 | 18.7 | 18.8 | 18.3 | 15.0 | 15.2 | 1.00 | 0.99 | 1.02 | 1.24 | 1.22 |
|  | GAG | 6.7 | 7.1 | 8.6 | 9.3 | 11.3 | 11.5 | 0.94 | 0.78 | 0.72 | 0.60 | 0.58 |
| Cys | UGU | 14.5 | 14.4 | 14.2 | 12.7 | 9.4 | 9.3 | 1.00 | 1.02 | 1.14 | 1.54 | 1.55 |
|  | UGC | 14.4 | 21.1 | 14.9 | 20.8 | 20.1 | 20.3 | 0.68 | 0.96 | 0.69 | 0.72 | 0.71 |
| TER | UGA | 20.9 | 20.6 | 16.9 | 18.1 | 15.4 | 15.8 | 1.01 | 1.23 | 1.15 | 1.36 | 1.32 |
| Trp | UGG | 10.8 | 19.8 | 13.9 | 18.6 | 18.2 | 19.4 | 0.55 | 0.77 | 0.58 | 0.59 | 0.56 |
| Arg | CGU | 7.4 | 10.3 | 10.0 | 15.8 | 14.5 | 14.8 | 0.72 | 0.74 | 0.47 | 0.51 | 0.50 |
|  | CGC | 5.4 | 13.5 | 8.4 | 24.4 | 34.1 | 34.2 | 0.40 | 0.64 | 0.22 | 0.16 | 0.16 |
|  | CGA | 8.5 | 10.7 | 9.1 | 15.5 | 17.8 | 18.8 | 0.79 | 0.93 | 0.55 | 0.48 | 0.45 |
|  | CGG | 4.2 | 14.1 | 6.9 | 18.8 | 26.4 | 26.8 | 0.30 | 0.61 | 0.23 | 0.16 | 0.16 |
| Ser | AGU | 13.1 | 11.9 | 13.4 | 10.8 | 7.9 | 7.8 | 1.10 | 0.98 | 1.21 | 1.67 | 1.69 |
|  | AGC | 15.5 | 17.7 | 15.5 | 17.2 | 22.2 | 22.3 | 0.87 | 1.00 | 0.90 | 0.70 | 0.69 |
| Arg | AGA | 16.9 | 13.3 | 13.5 | 12.1 | 11.0 | 11.0 | 1.28 | 1.26 | 1.40 | 1.54 | 1.55 |
|  | AGG | 11.8 | 11.4 | 9.4 | 10.9 | 13.3 | 13.7 | 1.04 | 1.26 | 1.08 | 0.89 | 0.86 |
| Gly | GGU | 9.5 | 16.8 | 13.9 | 16.4 | 16.5 | 17.0 | 0.56 | 0.68 | 0.58 | 0.57 | 0.56 |
|  | GGC | 7.8 | 18.2 | 10.2 | 20.2 | 28.5 | 29.4 | 0.43 | 0.76 | 0.38 | 0.27 | 0.26 |
|  | GGA | 9.5 | 10.5 | 8.1 | 12.1 | 13.6 | 13.7 | 0.90 | 1.17 | 0.78 | 0.70 | 0.69 |
|  | GGG | 4.3 | 8.1 | 5.8 | 10.4 | 13.7 | 14.6 | 0.53 | 0.74 | 0.42 | 0.32 | 0.30 |

**References**

M. Ahmad, H. Prensky, J. Balestrieri, S. ElNaggar, A. Gomez-Simmonds, A.-C. Uhlemann, B. Traxler, A. Singh and A.J. Lopatkin, Tradeoff between lag time and growth rate drives the plasmid acquisition cost, *Nature Communications* **14** (2023), p. 2343.

N.-F. Alikhan, N.K. Petty, N.L.B. Zakour and S.A. Beatson, BLAST Ring Image Generator (BRIG): simple prokaryote genome comparisons, *BMC genomics* **12** (2011), pp. 1-10.

N. Dong, Y. Zeng, C. Cai, C. Sun, J. Lu, C. Liu, H. Zhou, Q. Sun, L. Shu and H. Wang, Prevalence, transmission, and molecular epidemiology of tet (X)-positive bacteria among humans, animals, and environmental niches in China: An epidemiological, and genomic-based study, *Science of The Total Environment* **818** (2022), p. 151767.

M. El-Mansi, K.J. Anderson, C.A. Inche, L.K. Knowles and D.J. Platt, Isolation and curing of the Klebsiella pneumoniae large indigenous plasmid using sodium dodecyl sulphate, *Research in microbiology* **151** (2000), pp. 201-208.

T. He, R. Wang, D. Liu, T.R. Walsh, R. Zhang, Y. Lv, Y. Ke, Q. Ji, R. Wei and Z. Liu, Emergence of plasmid-mediated high-level tigecycline resistance genes in animals and humans, *Nature microbiology* **4** (2019), pp. 1450-1456.

K.A. Kleinheinz, K.G. Joensen and M.V. Larsen, Applying the ResFinder and VirulenceFinder web-services for easy identification of acquired antibiotic resistance and E. coli virulence genes in bacteriophage and prophage nucleotide sequences, *Bacteriophage* **4** (2014), p. e27943.

S. Koren, B.P. Walenz, K. Berlin, J.R. Miller, N.H. Bergman and A.M. Phillippy, Canu: scalable and accurate long-read assembly via adaptive k-mer weighting and repeat separation, *Genome research* **27** (2017), pp. 722-736.

M.M. Lam, J. Koong, K.E. Holt, R.M. Hall and M. Hamidian, Detection and typing of plasmids in Acinetobacter baumannii using rep genes encoding replication initiation proteins, *Microbiology Spectrum* **11** (2023), pp. e02478-02422.

I. Letunic and P. Bork, Interactive Tree Of Life (iTOL) v5: an online tool for phylogenetic tree display and annotation, *Nucleic acids research* **49** (2021), pp. W293-W296.

C. Liu, N. Dong, Y. Zeng, J. Lu, J. Chen, Y. Wang, C. Cai, K. Chen, G. Chen and Z. Shen, Co-transfer of last-line antibiotic resistance and virulence operons by an IncFIBk-FII-X3-ColKP3 hybrid plasmid in Klebsiella pneumoniae, *Journal of Antimicrobial Chemotherapy* (2022).

Z. Lu, X. Yuan, S. Li, Q. Shan and Y. Wang, Codon Usage Bias Analysis of Gerbera jamesonii Based on Transcriptome Sequences, *JOURNAL OF SOUTHWEST FORESTRY UNIVERSITY* **43** (2023), pp. 77-86.

S.C. Nang, F.C. Morris, M.J. McDonald, M.-L. Han, J. Wang, R.A. Strugnell, T. Velkov and J. Li, Fitness cost of mcr-1-mediated polymyxin resistance in Klebsiella pneumoniae, *Journal of Antimicrobial Chemotherapy* **73** (2018), pp. 1604-1610.

A.J. Page, C.A. Cummins, M. Hunt, V.K. Wong, S. Reuter, M.T. Holden, M. Fookes, D. Falush, J.A. Keane and J. Parkhill, Roary: rapid large-scale prokaryote pan genome analysis, *Bioinformatics* **31** (2015), pp. 3691-3693.

A. Pérez, M. Poza, A. Fernández, M. del Carmen Fernández, S. Mallo, M. Merino, S. Rumbo-Feal, M.P. Cabral and G. Bou, Involvement of the AcrAB-TolC efflux pump in the resistance, fitness, and virulence of Enterobacter cloacae, *Antimicrobial agents and chemotherapy* **56** (2012), pp. 2084-2090.

M.N. Price, P.S. Dehal and A.P. Arkin, FastTree 2–approximately maximum-likelihood trees for large alignments, *PloS one* **5** (2010), p. e9490.

Q. Pu, X.-T. Fan, A.-Q. Sun, T. Pan, H. Li, S.B. Lassen, X.-L. An and J.-Q. Su, Co-effect of cadmium and iron oxide nanoparticles on plasmid-mediated conjugative transfer of antibiotic resistance genes, *Environment International* **152** (2021), p. 106453.

T. Seemann, Prokka: rapid prokaryotic genome annotation, *Bioinformatics* **30** (2014), pp. 2068-2069.

N. Soler and P. Forterre, Vesiduction: the fourth way of HGT, *Environmental Microbiology* **22** (2020), pp. 2457-2460.

M.J. Sullivan, N.K. Petty and S.A. Beatson, Easyfig: a genome comparison visualizer, *Bioinformatics* **27** (2011), pp. 1009-1010.

J. Sun, R.-S. Yang, Q. Zhang, Y. Feng, L.-X. Fang, J. Xia, L. Li, X.-Y. Lv, J.-H. Duan and X.-P. Liao, Co-transfer of blaNDM-5 and mcr-1 by an IncX3–X4 hybrid plasmid in Escherichia coli, *Nature microbiology* **1** (2016), pp. 1-4.

E.C.o.A.S. Testing and E.C.o.A.S. Testing, Breakpoint tables for interpretation of MICs and zone diameters. Version 11.0, 2021. (2021).

Y. Wang, J. Lu, S. Zhang, J. Li, L. Mao, Z. Yuan, P.L. Bond and J. Guo, Non-antibiotic pharmaceuticals promote the transmission of multidrug resistance plasmids through intra-and intergenera conjugation, *The ISME journal* **15** (2021), pp. 2493-2508.

M.P. Weinstein, Performance standards for antimicrobial susceptibility testing, Clinical and Laboratory Standards Institute (2021).

Q.E. Yang, X. Ma, L. Zeng, Q. Wang, M. Li, L. Teng, M. He, C. Liu, M. Zhao and M. Wang, Interphylum dissemination of NDM-5-positive plasmids in hospital wastewater from Fuzhou, China: a single-centre, culture-independent, plasmid transmission study, *The Lancet Microbe* **5** (2024), pp. e13-e23.

R. Zhang, N. Dong, Z. Shen, Y. Zeng, J. Lu, C. Liu, H. Zhou, Y. Hu, Q. Sun and Q. Cheng, Epidemiological and phylogenetic analysis reveals Flavobacteriaceae as potential ancestral source of tigecycline resistance gene tet (X), *Nature communications* **11** (2020a), pp. 1-13.

R. Zhang, N. Dong, Y. Zeng, Z. Shen, J. Lu, C. Liu, Z.-a. Huang, Q. Sun, Q. Cheng and L. Shu, Chromosomal and plasmid-borne tigecycline resistance genes tet (X3) and tet (X4) in dairy cows on a Chinese farm, *Antimicrobial agents and chemotherapy* **64** (2020b), pp. e00674-00620.
